# Accurate, robust and harmonized implementation of morpho-functional imaging in treatment planning for personalized radiotherapy

**DOI:** 10.1371/journal.pone.0210549

**Published:** 2019-01-09

**Authors:** Elisa Jiménez-Ortega, Ana Ureba, José Antonio Baeza, Ana Rita Barbeiro, Marcin Balcerzyk, Ángel Parrado-Gallego, Amadeo Wals-Zurita, Francisco Javier García-Gómez, Antonio Leal

**Affiliations:** 1 Departamento de Fisiología Médica y Biofísica, Universidad de Sevilla, Seville, Spain; 2 Instituto de Biomedicina de Sevilla, IBIS, Seville, Spain; 3 Centro Nacional de Aceleradores (CNA), Universidad de Sevilla, Junta de Andalucía, Consejo Superior de Investigaciones Científicas (CSIC), Seville, Spain; 4 Hospital Universitario Virgen Macarena, Servicio de Radioterapia, Seville, Spain; 5 Hospital Universitario Virgen Macarena, Servicio de Medicina Nuclear, Seville, Spain; North Shore Long Island Jewish Health System, UNITED STATES

## Abstract

In this work we present a methodology able to use harmonized PET/CT imaging in dose painting by number (DPBN) approach by means of a robust and accurate treatment planning system. Image processing and treatment planning were performed by using a Matlab-based platform, called CARMEN, in which a full Monte Carlo simulation is included. Linear programming formulation was developed for a voxel-by-voxel robust optimization and a specific direct aperture optimization was designed for an efficient adaptive radiotherapy implementation. DPBN approach with our methodology was tested to reduce the uncertainties associated with both, the absolute value and the relative value of the information in the functional image. For the same H&N case, a single robust treatment was planned for dose prescription maps corresponding to standardized uptake value distributions from two different image reconstruction protocols: One to fulfill EARL accreditation for harmonization of [^18^F]FDG PET/CT image, and the other one to use the highest available spatial resolution. Also, a robust treatment was planned to fulfill dose prescription maps corresponding to both approaches, the dose painting by contour based on volumes and our voxel-by-voxel DPBN. Adaptive planning was also carried out to check the suitability of our proposal.

Different plans showed robustness to cover a range of scenarios for implementation of harmonizing strategies by using the highest available resolution. Also, robustness associated to discretization level of dose prescription according to the use of contours or numbers was achieved. All plans showed excellent quality index histogram and quality factors below 2%. Efficient solution for adaptive radiotherapy based directly on changes in functional image was obtained. We proved that by using voxel-by-voxel DPBN approach it is possible to overcome typical drawbacks linked to PET/CT images, providing to the clinical specialist confidence enough for routinely implementation of functional imaging for personalized radiotherapy.

## Introduction

Although it is well known that patients often have varied tumor responses to radiation therapy (RT) due to differences in tumor type and other genetic factors, dose prescription and treatment planning is essentially a population-based approach. Biological considerations are not included in the planning process except by means of mathematical models parametrized to predict the RT outcome [1]. More than any other treatment modality, success of RT depends on medical imaging because it is used for determining the disease extension and defining target region and healthy tissues for planning the treatment. Therefore, it is a clear example of theranostics in the field of oncology where diagnostics and therapeutics should be co-developed. Unfortunately, imaging in RT is usually limited to three-dimensional (3D) anatomical information from conventional computed tomography (CT) for dose prescription. A more ambitious approach, image guided RT (IGRT), allows including time as an additional dimension to minimize geometrical uncertainties in patient and tumor positions and volume during the course of the treatment. Physiological information could be provided by functional image modalities such as functional CT, magnetic resonance imaging (fMRI) and positron emission tomography (PET), in order to consider the radiation effect and to parametrize the disease evolution of each patient. Nevertheless, biological considerations are rarely included in the planning process, being patient individualized approach versus the conventional population-based approach a challenge still to be solved in RT. PET/CT combines biological activity and anatomical information in a single study session and it is the more recurrent functional modality in RT. Although it is widely considered for cancer staging [2], and even it was shown to be useful for target volume definition, especially for lung tumors [3], the implementation of PET/CT image information in the planning process is still challenging and present some practical limitations.

Dose painting (DP) strategies in RT [4] propose that, in order to maximize local tumor control, radiation dose should be “painted” based on the patient-specific tumor biological properties, in contrast to conventional RT, which aims to deliver a uniform dose to the whole tumor volume. DP was coined for referring the ability to further customize the delivered dose distribution based on the pertinent biological information derived from patient image. DP should be especially taken into account as a feasible approach for new hypofractionation schemes [5,6], in which the total dose of radiation is divided into larger doses in fewer treatment sessions than usual. Considering this trend in RT, planning based on CT scan at a single pretreatment time point to delineate the planning target volume (PTV) and organs at risk (OARs) is reviewed to potential anatomical changes and it should be also revised to physiological variations in order to apply the necessary adaptation during the course of the treatment. Nevertheless, imaging-based DP leads to the prescription and delivery of a non-uniform dose to the clinical target volume (CTV), what means a novel paradigm in RT and therefore, a clinical application slower than usual. Indeed, in spite of recent technology to improve spatial resolution and detection sensitivity, the inclusion of PET/CT data in the optimization process for planning is not as spread as expected. Anyway, biological target volume (BTV) is a new definition linked to DP in order to represent a subvolume of the tumor with specific characteristics on functional or molecular imaging technique, although there is controversial about whether this information can be adequately described with discrete volumes [7].

Apart from temporal resolution [8], spatial heterogeneity in biological characteristics such as hypoxia, proliferation or perfusion determine spatial variation in radiation sensitivity. This is dependent on spatial resolution involved in both, the image reconstruction and the grid used for dose calculation. Unlike the well-known procedure based on conventional morphological image from CT, some harmonization procedure based on accurate quantification and reproducibility is still required to make possible normalized prescription dose in multicenter studies [9,10] and also for implementing the biological information in the treatment planning systems (TPSs) in the ordinary way. On the other hand, the effort to establish uniform protocols for the harmonization of image quality and quantitative accuracy for multicenter clinical trials [11], such as EARL (ResEARch 4 Life, the accreditation granted by the European Association of Nuclear Medicine [12]), leads to image reconstructions involving parameters which are not taking advantage of the higher features of the latest scanners. This matter is not trivial since the selected reconstruction parameters strongly influence on the incorporation of intra-tumoral heterogeneity into treatment response assessment [13].

Even though a normalization protocol was adopted, the traditional volumes definition still could involve a discretization level inadequate to describe the actual gradients of oxygen tension or the density distribution of radioresistant cellular phenotypes [7]. Spatial resolution in PET scanner determines how far the signal spreads around its actual location, what is described by the point spread function (PSF). This invariable finite resolution generates so called partial volume effect (PVE) [14], resulting in an important uncertainty in PET tumor imaging. The necessary partial-volume correction methods depend heavily on the implemented volumes definition methods, so uncertainties in PET-based volumes propagate into uncertainties in correction methods such as the based on recovery coefficient, what can even worsen accuracy and precision [15]. Also, BTV definition is biased due to the necessary alignment of the different coordinate systems with different voxel sizes of anatomical and metabolic dataset.

All these uncertainties commented above, besides the extra workload and inherent associated cost, justify the mistrust linked to the PET/CT imaging implementation into the TPSs for clinical routine.

In order to overcome the uncertainties mentioned above regarding harmonization and discretization of biological targets, we present a methodology implemented in a robust TPS able to solve DP approach at voxel level for biological targeting and prescription of dose. This approach also provides the more accurate dose calculation based on Monte Carlo (MC) simulations and is ready for evaluation and fair comparison with traditional approach based on volumes.

## Materials and methods

### Treatment planning system for theranostics

For this work, morpho-functional image processing was performed by using a full Monte Carlo (fMC) TPS developed by our group, called CARMEN [http://grupos.us.es/medicalphysics], already used in previous works to calculate complex radiotherapy treatments [16–19]. CARMEN is a Matlab-based platform with parallel computing ability both multicore and GPU. Besides the algorithms for fMC planning, it provides users a toolset for the evaluation and management of medical images with complete control of calculation grid sizes, pre- and post-processing filters and interpolation methods. In this work, we use DICOM images from PET/CT with ^18^F-fluorodeoxyglucose ([^18^F]FDG). Nonetheless, this platform supports data from other medical imaging systems under DICOM protocol, or matrices corresponding to dose distributions or to digital information packed in the most used image file formats.

Once the image datasets are loaded in CARMEN platform, our approach can manage this information voxel by voxel along the whole process, i.e., from the definition of volumes of interest for prescription dose in the diagnostic stage up to the optimization and dose calculation of treatment planning in the therapeutic stage.

Regarding the dose calculation process, the expected dose distribution is computed in the patient image, voxelized with radiological information from CT for considering the particles transport in beam through different media, basically air, bone, lung, and normal tissue. In order to improve the accuracy of this calculation, some advanced commercial solutions already implement MC to calculate the dose distribution in the voxelized image of patient. For this work, due to the potential influence of the scatter radiation from beam modifiers on the high heterogeneity expected in the dose, transport through the collimation components such as multi-leaf collimator (MLC), was also considered. Thus, the explicit transport of the particles in the beam was followed through the linac head, considering the beam modifiers in the planning process. We used BEAMnrc [20] code based on EGSnrc [21] for the simulation of the linac head and BEAMDOSE [22], a DOSXYZnrc code [23] modification, to consider the dose deposition in each voxel of the patient CT.

### Harmonization procedure

As it was mentioned above, DP requires repeatability and reproducibility in PET acquisition settings and data analysis methods in order to be able to normalize the prescription dose. For a previous work we achieved the EARL European accreditation for the PET/CT scanner used in this study [24], in order to obtain the needed precision and accuracy for a consensual prescription of the dose. After multiple measurements with the image quality phantom NEMA 2–2007 [25], we established the image reconstruction protocol able to achieve the EARL accreditation. The applied reconstruction type was OSEM3D (Ordered Subset Expectation Maximization in three dimensions) with 21 iterations and 21 subsets, with a voxel size of 3.2 x 3.2 x 5 mm^3^, a grid of 256 x 256 pixels, and a post-processing Gaussian filter with 6 mm of width. Unfortunately, this protocol tries to normalize devices with different technical specifications, so in new scanners like the one used for this work, a Siemens Biograph mCT 64 [26], some features in terms of image resolution and quantification, are unexploited. In order to maintain the highest resolution of our scanner, what is important for the best volumes definition, another protocol for image reconstruction, named BIOGRAPH, was carried out. In BIOGRAPH protocol, the reconstruction type was PSF with 21 iterations and 21 subsets, with a voxel size of 1.6 x 1.6 x 1.5 mm^3^, a grid of 512 x 512 pixels, and a post-processing Gaussian filter with 3 mm width. CT-based attenuation and time of flight correction method were applied in the two protocols.

As an example, an actual clinical case was selected for this work with head and neck (H&N) cancer with small targets and also low activity in some regions. This study was reviewed and approved by the Andalusian public foundation for health research management in Seville (FISEVI). This institution was the entity to manage the public funding received by the Junta de Andalucía for the project CTS-2482, in which this work is framed. The solutions for this actual case selected as a proof of concept were assessed only over the patient data adequately anonymized and ethic committee waived the need for any additional consent since no clinical application was carried out on the patient based on the study presented here.

**Fig 1:**
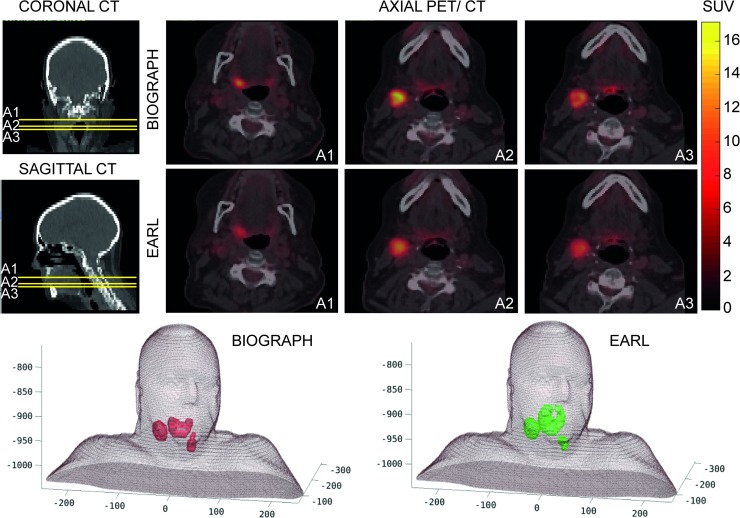
Different segmentation results following two image reconstruction protocols from PET/CT study of the same H&N case. Upper rows show three axial slices (A1, A2, A3) of PET/CT images with BIOGRAPH protocol based on a reconstruction keeping the highest resolution available by using our Siemens Biograph mCT 64 scanner and for EARL protocol based on a reconstruction to fulfill the EARL European accreditation. Lower row shows a 3D visualization of the biological target volumes by means of the same segmentation algorithm on the PET/CT image reconstructions from BIOGRAPH (red) and EARL (green) protocols.

The corresponding quantification for this complex case was highly dependent on the protocol of acquisition and reconstruction of the image. Fig 1 shows how significant can be the variability in standardized uptake value (SUV) quantification between both [^18^F]FDG PET/CT image reconstructions obtained with the same scanner. For BTs definition, the same algorithm for semi-automatic segmentation, based on affine propagation, was used [27], which takes into account the diffuse characteristic and multifocal nature of the PET image. In the 3D visualization of Fig 1, the differences between volumes can be seen, where reconstruction effects, such as PVE, lead to SUV distributions with no scalable factor between them.

It would be desirable to achieve a solution that meets with the requirements established by the EARL accreditation and that also allows to use the best available resolution for the targets definition. For it, the information of the images from both reconstructions would have to be implemented in a unique optimization process. However, if the biological targets are managed as volumes, it is not possible to assess which region of what target is satisfied with the corresponding dose prescription. The same can be said for clustering of voxels. At least, a compromise solution could be achieved by considering the dose prescription at the voxel level into an adequate optimization algorithm able to impose one or another prescription as appropriate to fulfill the global objective. In order to provide a solution to this problem, we implemented a linear programming (LP) formulation in our planning process [18]. LP based optimization allows to keep the molecular information from the functional image throughout the planning process, as demonstrated in a recent study [19]. The mathematical expression of the new algorithm is presented below (1). LP method has been modified to achieve a compromise solution at the voxel level, where the dose restrictions are within the prescribed values in each voxel for both reconstructions (EARL and BIOGRAPH).

$$OF\equiv P_{T,max}\sum_{i=1}^{N_{T}}x_{i}+P_{T,min}\sum_{i=1}^{N_{T}}y_{i}+P_{OAR,max}\sum_{i=N_{T}+1}^{N}x_{i}$$

subject to

$$\sum_{j=1}^{M}\omega_{j}d_{ij}-x_{i}\leq D_{i,max}\Delta_{i,max}\;i=1,\ldots ,N_{T}$$

$$\sum_{j=1}^{M}\omega_{j}d_{ij}+y_{i}\geq D_{i,min}\Delta_{i,min}\;i=1,\ldots ,N_{T}$$

$$\sum_{j=1}^{M}\omega_{j}d_{ij}-x_{i}\leq D_{OAR,max}\;i=N_{T}+1,\ldots ,N$$

$$x_{i},\;y_{i},\;\omega_{j}\geq 0\;\forall i,j$$

This algorithm tries to minimize the objective function (*OF*), where the overdose and underdose in each voxel *i* of the target (*x*_*i*_ and *y*_*i*_, respectively) are penalized by the factors *P*_*T*,*max*_ and *P*_*T*,*min*_ respectively, and the overdose in each voxel *i* of the OARs (*x*_*i*_) is penalized by the factor *P*_*OAR*,*max*_. *N*_*t*_ is the total number of voxels of the target and *N* is the total number of voxels of the problem. Moreover, each voxel of the target must fulfill the restrictions of maximum and minimum dose (*D*_*i*,*max*_ and *D*_*i*,*min*_) imposed according to the information in the functional image for both reconstruction methods, with similarity factors (*Δ*_*i*,*max*_ and *Δ*_*i*,*min*_) that allows a robust response to different dose prescriptions with the same treatment for each patient. *ω*_*j*_ is the weight of intensity for each aperture of the RT treatment, *d*_*ij*_ the dose deposited by each aperture in each voxel, and M the total number of apertures. *D*_*OAR*,*max*_ is the dose restriction for the OARs and depends on the toxicity of each one.

### Co-registration of image dataset and calculation grid size

During the planning process, the dose calculation is carried out by using a grid size usually different to the PET/CT grid size, which is also the result of a co-registration process. Although the size of the CT grid is 512 x 512 pixels per axial slice, the usual size for the dose calculation grid in the commercial TPSs is 128 x 128 pixels, or even 64 x 64 pixels per axial slice in order to minimize the computational time. Our proposal maintains a commitment to precision so, to account for the expected high heterogeneity, a grid size of 256 x 256 voxels was used in the dose calculation. In order to test the more adequate co-registration for the grid of dose calculation, three different interpolation methods of the image dataset were analyzed with CARMEN platform: three-dimensional linear, nearest-neighbor and cubic spline.

**Fig 2:**
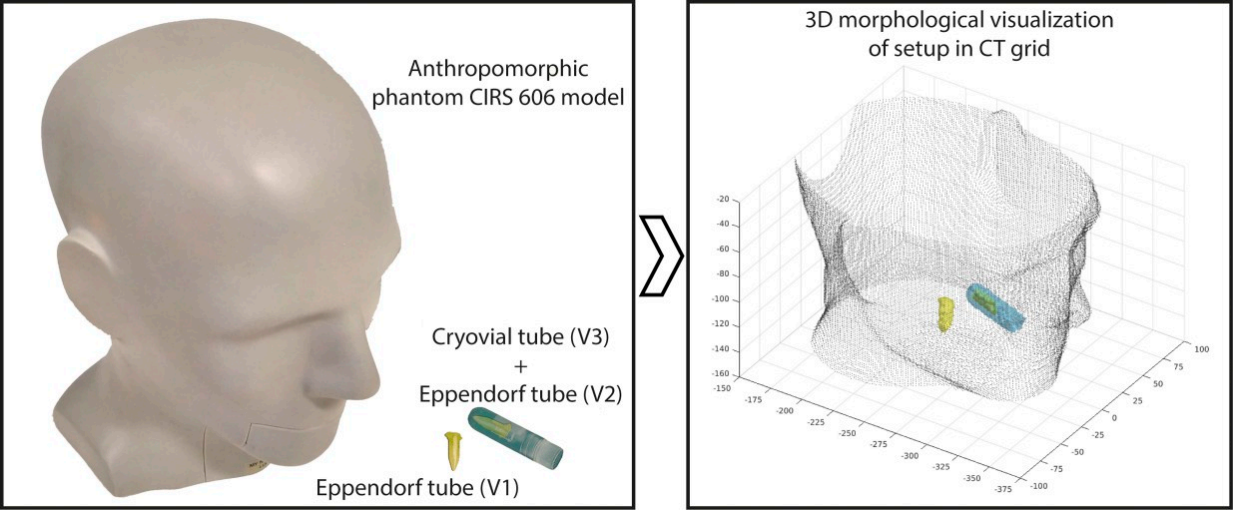
Experimental configuration for co-registration study in the theranostic process. On the left, phantom and tubes with different volumes and activities of [^18^F]FDG taken as real reference for being reconstructed from a PET/CT scan. On the right, the corresponding segmentation of volumes for 3D visualization in the CT grid.

Fig 2 presents the experimental setup for this co-registration study. At the left side, a picture of the anthropomorphic phantom (CIRS 606 model) is shown, which contains tissue simulating resins that mimic the X-ray attenuation properties of human tissue in head and neck. Lesions with different volumes and uptake values were simulated to perform realistic SUV segmentations with the highest possible resolution. The left side of Fig 2 also shows the Eppendorf tube (V1) of 0.3 ml, which was filled with [^18^F]FDG, with 0.116 MBq of activity, and located inside the phantom to simulate a lesion with small size in the area of the larynx. Another Eppendorf tube (V2), equal to V1, was introduced inside a Cryovial tube (V3) with 2 ml of [^18^F]FDG and 0.1 MBq of activity, which was also hosted in the phantom. This last set, V2+V3, intended to simulate a lesion with heterogeneous uptake in the oral cavity and, at the same time, represents a different scenario for the Eppendorf tube.

The phantom was scanned with the usual positioning for head and neck cases in RT, and the PET/CT images were reconstructed following both, the EARL and BIOGRAPH protocols. The two sets of PET/CT images were segmented using the above mentioned affine propagation algorithm [27]. Due to the lack of background SUV, it was possible to study the active volumes with the two image reconstructions methods and their correspondence with the known actual volumes. The direct comparison between segmented and actual volumes clearly shows that the EARL protocol led to wrong volume reconstruction, as it was expected. Therefore, for the image from BIOGRAPH protocol, only the most appropriate procedure was selected to be implemented in this study. According the data in Table 1, the interpolation method selected for the PET and CT fusion images was cubic spline interpolation, and nearest-neighbor interpolation for resampling these data in the dose calculation grid size. The choice was taken by considering the lower deviation for all volumes in the different scenarios for each stage along the process. As it was expected due to the size of actual volumes, smaller than the recommended for PET/CT application, the deviations shown in Table 1 are large. Nevertheless, these small volumes were selected for this test, in order to assess the procedure under an adequate 'stress-proof'.

**Table 1:** PET/CT quantification of known volumes with different activity of 18F-FDG inside an anthropomorphic phantom.

PET grid → CT grid					CT grid → Dose calculation grid				
Interpolation method	V1 (% deviation from the real 0.30 ml)	V2 (% deviation from the real 0.30 ml)	V3 (% deviation from the real 2.00 ml)	V2 + V3 (% deviation from the real 2.30 ml)	Interpolation method	V1 (% deviation from the real 0.30 ml)	V2 (% deviation from the real 0.30 ml)	V3 (% deviation from the real 2.00 ml)	V2 + V3 (% deviation from the real 2.30 ml)
Linear	0.38 (+26.7)	0.40 (+33.3)	1.96 (-2.0)	2.36 (+2.6)	Linear	0.35 (+16.7)	0.42 (+40.0)	2.21 (+10.5)	2.63 (+14.3)
					Nearest-neighbor	0.38 (+26.7)	0.40 (+33.3)	1.94 (-3.0)	2.34 (+1.7)
					Spline	0.64 (+113.3)	0.50 (+66.7)	2.43 (+21.5)	2.93 (+27.4)
Nearest-neighbor	0.34 (+13.3)	0.41 (+36.7)	1.86 (-7.0)	2.27 (-1.3)	Linear	0.31 (+3.3)	0.45 (+50.0)	1.95 (-2.5)	2.40 (+4.3)
					Nearest-neighbor	0.32 (+6.7)	0.36 (+20.0)	1.85 (-7.5)	2.21 (-3.9)
					Spline	0.60 (+100.0)	0.53 (+76.7)	2.34 (+17.0)	2.87 (+24.8)
Spline	0.36 (+20.0)	0.35 (+16.7)	1.91 (-4.5)	2.26 (-1.7)	Linear	0.31 (+3.3)	0.38 (+26.7)	2.09 (+4.5)	2.47 (+7.4)
					Nearest-neighbor	0.35 (+16.7)	0.33 (+10.0)	1.91 (-4.5)	2.24 (-2.6)
					Spline	0.60 (+100.0)	0.43 (+43.3)	2.41 (+20.5)	2.84 (+23.5)

Evaluation of different combination of interpolation methods for co-registration of image dataset from PET (1.6 x 1.6 x 1.5 mm voxel size)/CT (1.52 x 1.52 x 1.5 mm^3^ voxel size) with 512x512 pixels per slice and the dose calculation grid with 256x256 pixels per slice.

### Discretization level for biological image management

As it was commented before, BT definition by means of volumes implies the inclusion of uncertainties along the planning process and, therefore, biases the correct ulterior monitoring of disease. In response to this, DP has been proposed under two approaches: DP by contour (DPBC), where intensity thresholding of subvolumes within the conventional target is made and DP by number (DPBN), where the dose is prescribed directly to the values (numbers) in the functional image. Actually, DPBN approach could be considered as "true dose painting" in the sense that imaging can be really used for prescribing biological distributions and be implemented in the treatment planning algorithms for theranostics purpose. Unfortunately, as far as we know, there is no commercial TPS in which the numbers are directly managed. Traditionally, DPBN has been only used for specific research studies where the information was discretized by clustering of voxels, which is a halfway solution of the real challenge to incorporate the functional information in the optimization algorithm for the treatment planning. Clustering method remains an optimization based on dose to volumes so, the prescribed dose is done to a whole region as usual, by keeping the dose to healthy tissue under restrictions obeying toxicity levels imposed by population evidence-based medicine. On the other side, basing the optimization on the biological information provided by the numbers from the functional image supposes a change of paradigm that generates lack of confidence in the physicians. It would be desirable to have a tool for delineation and planning optimization at the voxel level, but which was also able to allow the traditional evaluation of the treatment based on dose to volumes.

Attending to this, we tried to reach a compromise solution for the same H&N case studied for the harmonization solution that would also allow establishing the degree of discretization for BT management. This solution would allow to carry out the true DPBN approach and to provide, at the same time, a directly transferable solution to the scenario as a result of using volumes. This joint evaluation was only possible by means of the optimization carried out through our LP formulation, since it is possible to prescribe the dose to each voxel, although this prescription can be the same for all the voxels corresponding to a specific clustering, what is defined for DPBC option. Now, in the algorithm, each voxel of the targets must fulfill the restrictions of maximum and minimum dose (*D*_*i*,*max*_ and *D*_*i*,*min*_), where each one of these limits is sometimes imposed by the dose prescription map corresponding to DPBC and other times by the corresponding to DPBN.

These dose prescription maps were imposed voxel by voxel through a linear relationship with SUV, what is usually applied for [^18^F]FDG [28]. If another radiotracer is used, such as an indicator of hypoxia or cell proliferation, other relations to impose the prescription map could be considered, but this methodology could be directly applicable.

### Adaptive radiotherapy

ART is the most important effort in RT for patient-tailored planning, and DP is the way to achieve the most ambitious adaptation of the treatment to the evolution of the disease. In this case, in order to provide a non-uniform radiation dose distribution to the intra-tumor heterogeneity, the most adequate technique is intensity modulated RT (IMRT), where variable radiation intensity is generated across multiple beams shaped with MLC sequenced during radiation delivery. Usually, a mathematical solution is achieved by means of an inverse method to obtain the map of intensity values for each beam, previously subdivided into virtual beamlets with an individual intensity. Segmentation and sequencing processes are used for finding the required geometrical MLC apertures able to provide the corresponding intensity maps. Therefore, the solution of the optimization process is a set of apertures for each incidence angle with different beam intensities. Unfortunately, searching the MLC apertures from intensity maps demands to start the replanning process from the beginning by considering the data image as a new case, what is a time-consuming process.

To overcome the inadequate optimization method based on the traditional intensity maps when biological considerations have to be taken into account, we developed the BIOMAP algorithm [18], already implemented in CARMEN, which is based on a specific direct aperture optimization assumed to be more efficient for ART [29]. This algorithm takes into account both the morphological and the functional information by means of the generation of ray-tracing projections for each incident beam, which are managed as matrices under Boolean combinations. Therefore, the sequencing process to obtain apertures is performed on maps with biophysical information instead of on intensity maps [18].

Because the apertures generated by BIOMAP are only based on the image information, possible changes in an updated image from IGRT or from follow-up PET image during the treatment could be easily incorporated in the planning, giving the mentioned patient-tailored treatment. This solution was integrated in our DP approach based on LP to manage the optimization at the voxel level.

For this work, we applied our approach to solve a potential adaptive planning of a virtual follow-up data image of the same H&N case considering changes in BT due to the calculated dose, assuming that it was the received dose. No morphological changes were included, but it did not affect the presented methodology since our algorithm can consider each voxel for dose calculation with the corresponding new physical density and SUV for dose prescription. Initially, this case presented a BT composed by three separate lesions to receive a prescription dose of 70 Gy during 30 sessions and a dose escalation up to 82 Gy inside the follow-up SUV heterogeneity.

### Evaluation methods

The cumulative dose volume histogram (DVH) represents the relative volume of the structures receiving doses greater than or equal to values discretized in bins. As in DPBN there are no such volumes, a new evaluation of dose different from the DVH has to be considered. We used the quality index (Q) [28], which is defined as the ratio between the planned dose and the prescribed dose in each voxel and thus, the unity is considered as the ideal value. Usually, this index is visualized as a histogram (QVH) to represent the results in a way closer to the traditional evaluation. Another criterion is the quality factor (QF), defined as the average absolute deviation of Q from 1 within BTs.

Histogram methodology does not provide any spatial information, so the dose evaluation should include the visualization of isodose lines over the image to show the relative dose distribution covering the targets and avoiding OARs as far as possible. Another usual parameter for evaluation is the conformity index (CI) defined as the ratio between the volume covered by the reference isodose (usually, 95% of prescription dose) and the whole target volume. In this work we did an effort to present results based on numbers in the way that the planning solutions based on volumes are usually evaluated, in order to provide a fair comparison.

## Results

### Solution for targets defined with the available highest resolution and under harmonizing requirements

In Table 2, we compare the results obtained with our DPBN proposal corresponding to three different plans for two dose prescription maps of the selected H&N case: P1 and P2 are the best plan solutions obtained for fulfilling the prescription maps to BTs segmented from the image reconstruction under BIOGRAPH and EARL protocol, respectively. P3 is a compromise plan able to satisfy both prescription maps, simultaneously. None of the plans did reach the toxicity levels to OARs. Although Q criteria is usually accepted when 90% of voxels is within a 5% of tolerance around the unit, percentage of voxels fulfilling the criteria with 3% and 4% were also obtained in order to assess the trend of our solutions.

**Table 2:** Quality evaluation of plans to the H&N case under European protocol (EARL) and the own protocol (BIOGRAPH) with the highest available resolution.

	Image protocol for generating the prescription map	P1	P2	P3
0.97<Q<1.03	BIOGRAPH	88.4%	73.5%	80.4%
	EARL	64.8%	84.2%	82.8%
0.96<Q<1.04	BIOGRAPH	93.0%	83.0%	90.9%
	EARL	71.7%	91.6%	89.3%
0.95<Q<1.05	BIOGRAPH	97.0%	90.9%	95.1%
	EARL	74.1%	96.1%	93.3%
QF	BIOGRAPH	1.6%	2.5%	1.8%
	EARL	9.4%	1.7%	1.9%

Percentage of voxels in BTs with quality index (Q) within 3%, 4% and 5% around 1 and the quality factor (QF) corresponding to the three plans (P1, P2 and P3) evaluated over the two dose prescription maps from both reconstruction protocols.

### DPBN solution at voxel level under the usual evaluation based on volumes for clinical application

**Fig 3:**
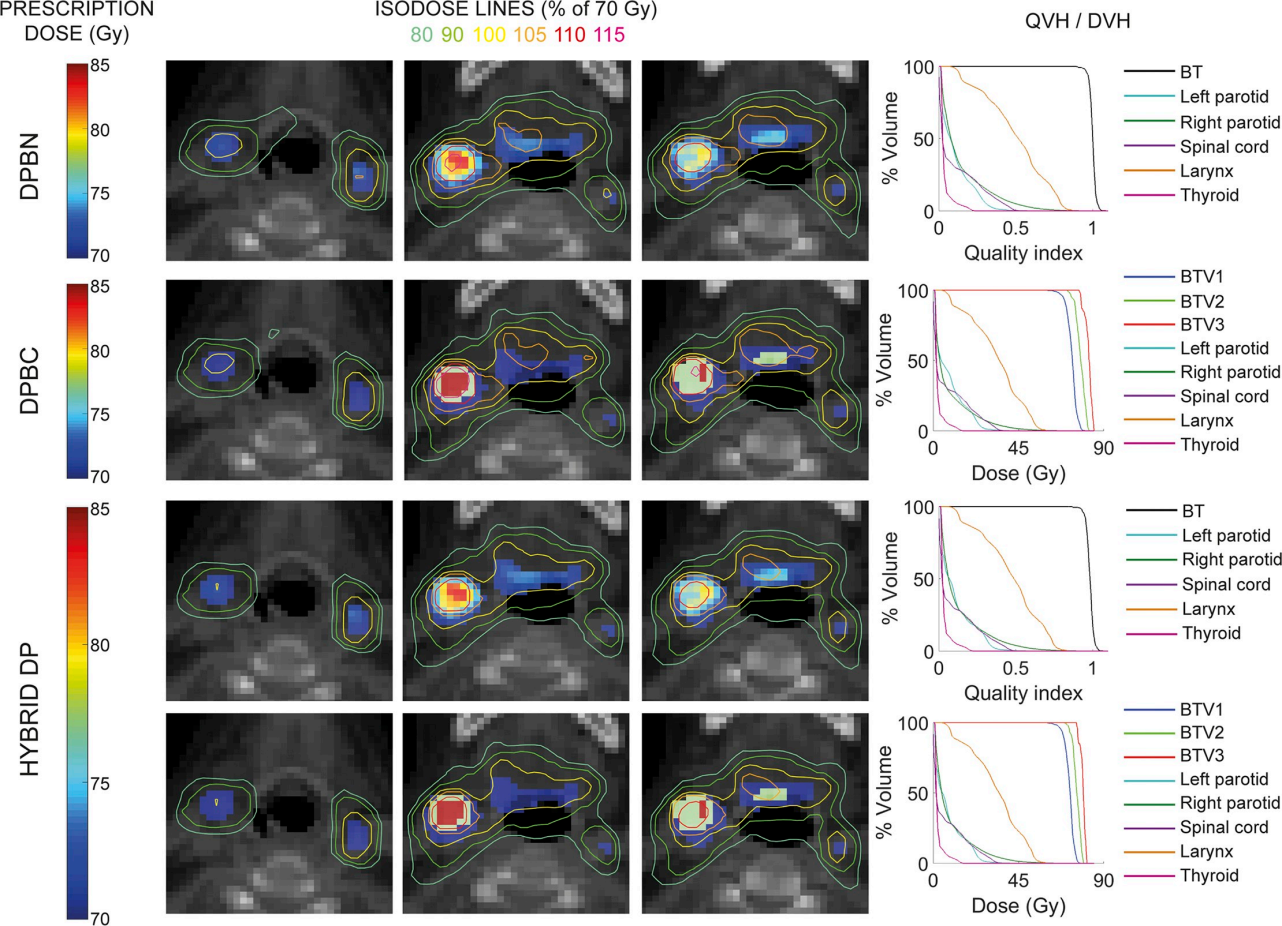
DPBN and DPBC plans to solve dose prescriptions based on numbers and on volumes, respectively, and HYBRID DP planning to solve both dose prescriptions. The corresponding isodose lines show the relative dose distributions as a percentage of the minimum prescription value (70 Gy) for the three plans. The isolines are visualized over both prescription approaches on CT image. DVHs and QVHs are presented at the left for each planning solving the corresponding dose prescription.

In Fig 3, we present evaluation results from the dose distributions of three different plans obtained for the same H&N case: DPBN plan to reach the prescription map based on numbers (BT); DPBC plan to reach the prescription doses to three BT volumes (BTV1, BTV2 and BTV3); and HYBRID DP plan able to respond to both prescriptions, simultaneously. Isodose lines are presented for three representative axial slices to meet both prescriptions represented on the CT images. Also, 3D evaluation based on DVH and QVH are presented. The first and second rows show the solutions for DPBN and DPBC to fulfill the corresponding prescriptions. The third and fourth rows show the robust solution fulfilling both prescriptions, simultaneously.

Quality evaluation results for the same three plan solutions are given in Table 3, regarding the Q, QF, and also the CI for the dose to volumes evaluation.

**Table 3:** Quality evaluation of plan solutions to achieve the dose prescription distribution based on DPBN and DPBC, and the robust solution (HYBRID DP).

	0.97<Q<1.03	0.96<Q<1.04	0.95<Q<1.05	QF	BTV1 (CI)	BTV2 (CI)	BTV3 (CI)
DPBN	88.4%	93.0%	97.0%	1.6%	-	-	-
DPBC	-	-	-	-	98.2%	96.0%	99.5%
HYBRID DP	84.1%	91.6%	94.7%	1.8%	95.7%	96.0%	88.0%

Percentage of voxels in BTs with quality index (Q) within 3%, 4% and 5% around 1, the quality factor (QF) and the conformity index (CI) are included.

### A solution for adaptive radiotherapy based on DPBN

**Fig 4:**
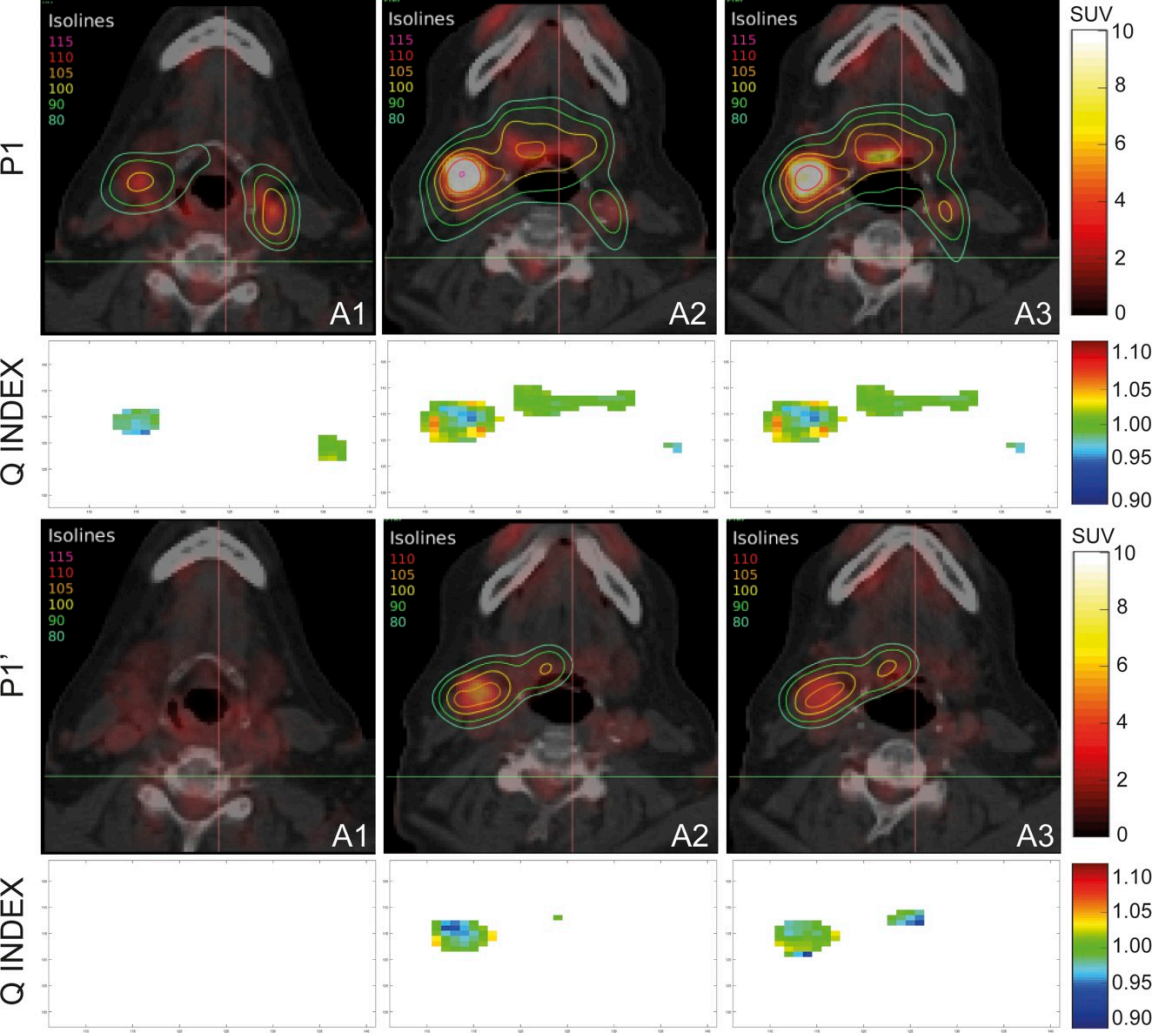
Representative isodose lines as a percentage of the minimum prescription value (70 Gy) corresponding to the plan solutions P1 and P1’ with our DPBN for prescription from initial and follow-up PET/CT image data, respectively. Q index representation is also included.

In Fig 4, we present isodose lines corresponding to the plan based on DPBN (P1) to solve the prescription map from the initial PET/CT scan (Phase I), and the isolines corresponding to the adaptive solution (P1’). This adaptive plan was obtained with the LP optimization of a set of apertures of only new few geometries (Table 4) provided by the application of BIOMAP algorithm on the new prescription map from the follow-up PET/CT scan (Phase II). Considering that isolines distribution is a relative dose evaluation method suited to volumes but not so much for numbers, as it is evident in Fig 3, we decided also to show in Fig 4 the Q index maps corresponding to the same slices for both, the initial planning to Phase I, and the adaptive planning to Phase II. Recurrent green color in these voxels maps corresponds to Q index equal to one. The shown isolines correspond to several percentages of the minimum prescription dose of 70 Gy and they covered the heterogeneous prescription according to both SUV maps.

**Table 4:** Quality evaluation of plans of the H&N case along the different phases of adaptive treatment.

	Phase I (Fractions 1–17)	Phase II (Fractions 18–30)	Total treatment
0.97 < Q < 1.03	88.4	83.5	86.7
0.96 < Q < 1.04	93.0	91.0	92.3
0.95 < Q < 1.05	97.0	95.5	96.5
QF	1.6%	1.8%	1.6%
Number of MLC apertures	130	43	

Table 4 presents Q index under different tolerance criteria and QF values for the two obtained adaptive plan solutions along treatment time. Number of necessary MLC apertures to solve the adaptive planning was also included.

## Discussion

### Solution for targets defined with the available highest resolution and under harmonizing requirements

The results presented on Table 2 revealed that when the dose prescription map evaluated was the one based on BIOGRAPH protocol, P1 solution was always of superior quality, showing larger percentage of voxels within the tolerance for Q, as expected. Similarly, P2 showed the same trend when the map evaluated was the one based on the EARL protocol. The robust solution P3 was slightly worse than P1 and P2 for BIOGRAPH and EARL protocols, respectively, but it was a good solution for both. The same can be said about QF. This trend was coherent with the level of tolerance, what is showing the precision of the methodology. It is important to remark that, to the best of our knowledge, P3 results showed higher percentage for Q and better QF than other published works [30–34], in spite of this solution was trying to satisfy two prescription maps simultaneously. Actually, our results of Q with 3% tolerance were comparable to previous published Q values with 5% tolerance, and our QF results were under 2%, when the usual criterion is 5%. Moreover, the significant low percentage of voxels within the tolerance when P1 and P2 were evaluated against the non-corresponding dose prescription maps, helps to show the high accuracy of the proposed methodology.

This robust solution satisfies the prescription based on the necessary harmonized image for multicenter studies and, simultaneously, fulfills the prescription corresponding to the available map with the highest resolution for the best biological target definition.

### DPBN solution at voxel level under the usual evaluation based on volumes for clinical application

The evaluation results of dose distribution based on volumes, from the isolines on Fig 3, showed effective coverage of the targets for all solutions, even for the hybrid approach, so this plan solved at the same time the case prescribed by numbers and by volumes, being this our main objective in this section. The corresponding 3D evaluation showed also that the solutions did not exceed the toxicity levels for OARs. In this way, this hybrid solution could allow using true DPBN approach for the best biological consideration in the patient image and also provides the specialists with the more usual evaluation of the solution.

As it could be seen from quality evaluation (Table 3), HYBRID DP did not provide results as good as those obtained with DPBN and DPBC for their corresponding prescriptions, but yet it covered enough both set of requirements. Again, we have to note that not only DPBN and DPBC solutions showed better results than in other works focused to DP, but also the hybrid solution achieved Q index and QF values superior to solutions already published. A similar trend of CI values was found for the hybrid solution. BTV3 showed a CI slightly lower than 90% for hybrid solution due to this volume was very small. Moreover, this methodology based on LP to carry out our DPBN approach allowed put together all data in the last row of Table 3, i.e., the direct comparison between both DP approaches for a fair comparison.

This methodology provided robust solution fulfilling both dose prescription maps, the one based on volumes and the one based on voxels. The latter allowed using the more exhaustive voxel-by-voxel planning process and, at the same time, the traditional evaluation based on volumes. In this work we showed that DPBN approach at the voxel level, implemented under the adequate method, is the best way to support the planning process from the biological target definition up to the optimization of heterogeneous dose distribution.

Although the results obtained by means the proposed methodology show an efficient way to overcome the uncertainties mentioned regarding harmonization and discretization of biological targets in a robust way, these results could be considered only a theoretical solution far away of the clinical application. In order to prove the feasibility of these solutions, we performed an experimental verification based on a model [35] involving a high demanding spatial resolution in dose distribution to test the dose prescription heterogeneity presents in DPBN (see S1 Fig).

### A solution for adaptive radiotherapy based on DPBN

Recent works showed the potential of ART for anatomy changes in order to reduce uncertainties arising from non-rigid setup errors and anatomy deformations, even by means of integrated adaptive solutions such as MRI-guided radiation therapy [36]. Although some centers are trying to clinically implement online adaptation, i.e., during the course of a treatment session, most efforts are being made to recruit information from in-room imaging, in order to identify patients likely to require ART in the remaining sessions by means of predictive models [37]. All these studies involve development of dose accumulation and strategies based on deformable image registration (DIR) for the alignment of datasets between the different image scans to consider anatomical changes extra to translations and rotations. The parametrization of these changes allows calculating systematic deformations in order to design strategies to overcome possible loss of target coverage by means of planning CT modifications [38] or by the definition of new margins around the PTV for robust planning [39]. These works are welcome to achieve the fundamental objective of individualized RT, but all of them are blind to molecular changes related to biological processes.

Repopulation of viable cells and clearance of dead cells due to radiation induced cellular damage are expected to increase with dose escalation schemes, so tumor responses such as shrinkage, are the result of both, anatomical and metabolic changes. Early treatment response assessment derived from biological information acquired during the course of treatment, gives an opportunity to deliver higher-dose radiation to the more aggressive areas of the tumor [40]. Only few studies considering biological changes in ART have been reported [30–32,41]. These works found sufficient changes in target volumes during the treatment to justify replanning, but the process to parametrize these changes via DIR involved even more complexity than those found when DIR is applied to anatomical changes, because of the variations between biological entities are too large. It does not seem that this can be solved while continuing to consider volumes in the way that has been done with morphological structures for planning.

The results obtained in this work from the evaluation of the adaptive solutions based on true DPBN for different treatment phases (Phase I and II), indicated the feasibility of our model to achieve solutions able to cover the heterogeneous prescription according to corresponding SUV maps, as it was shown in Fig 4 by the isolines, and also from the quality index results from Table 4. Some therapeutic isolines disappeared in P1’ solution because of some regions in follow-up image stopped showing metabolic activity. In fact, a window/level operation had to be applied to PET images in order to enhance them enough for the visualization of the follow-up SUV map, what caused saturation in the initial SUV map. In similar scenario, other works showed a color code for follow-up images different to the code used for initial image, perhaps because they worked attending only to the relative distribution and not to the absolute value. We think that the same code should be used once the harmonization is achieved.

It is also important to remark that the evaluation of the total treatment based on voxels, included on Table 4, was direct and a DIR algorithm was not necessary to express the dose summation to a non-rigid volume deformed along the treatment. This is a clearly different process involving fewer uncertainties if compared the methodology followed in other works to include the functional information in ART [31]. Moreover, the low number of new apertures or MLC geometries necessary to solve the adaptive planning (Table 4), showed to be in accordance to the methodology implemented in our BIOMAP algorithm, which is based exclusively in the image data.

## Conclusions

In this work, a methodology able to implement the morpho-functional image dataset in the whole RT planning process, from the biological target definition up to the dose calculation and evaluation was developed. We showed that DPBN approach at the voxel level is possible to overcome typical drawbacks linked to PET/CT images, providing to the clinical specialist confidence enough for routinely implementation of functional image in the planning process. The proposed algorithm for DPBN approach was able to provide robust solutions by means of the parameterization of uncertainties and the optimization based on dose prescriptions to targets and restrictions to OARs at the voxel level. In this way, DPBN approach can implement the functional information in the planning process, in order to take into account the biological considerations for radiation treatment, and to achieve really personalized treatments for cancer.

LP methodology proposed in this work for treatment planning optimization can be extended to images based on other biomarkers and/or acquired with other devices providing functional information. Also, the algorithm is extensible to other delivery systems as tomotherapy or protontherapy, as long as we consider the MC characterization of the corresponding beamlets as calculation units.

## Supporting information

S1 Fig Experimental verification with QuAArC system [35] of the dose distribution for the DPBN treatment for the head and neck case. (PDF)
