# Supplementary material for: Accurate, robust and harmonized implementation of morpho-functional imaging in treatment planning for personalized radiotherapy
Source: PLoS One. 2019 Jan 9;14(1):e0210549. doi: 10.1371/journal.pone.0210549 (PMC6326505; doi:10.1371/journal.pone.0210549)

S1 Figure. Experimental verification with QuAArC system (35) of the dose distribution for the DPBN treatment for the head and neck case.

S1A Figure. Gamma analysis. In the left, the dose distribution measured in the radiochromic film, once unrolled out of the cylindrical phantom. In the center, the dose distribution theoretically calculated with full Monte Carlo corresponding to the location where the radiochromic film is hosted inside the phantom. The shown dose values are the relative value against the minimum prescription dose (70 Gy). On the right, the gamma analysis between both dose distributions. Positive test corresponding to blue color was achieved in the 96.2% of points.

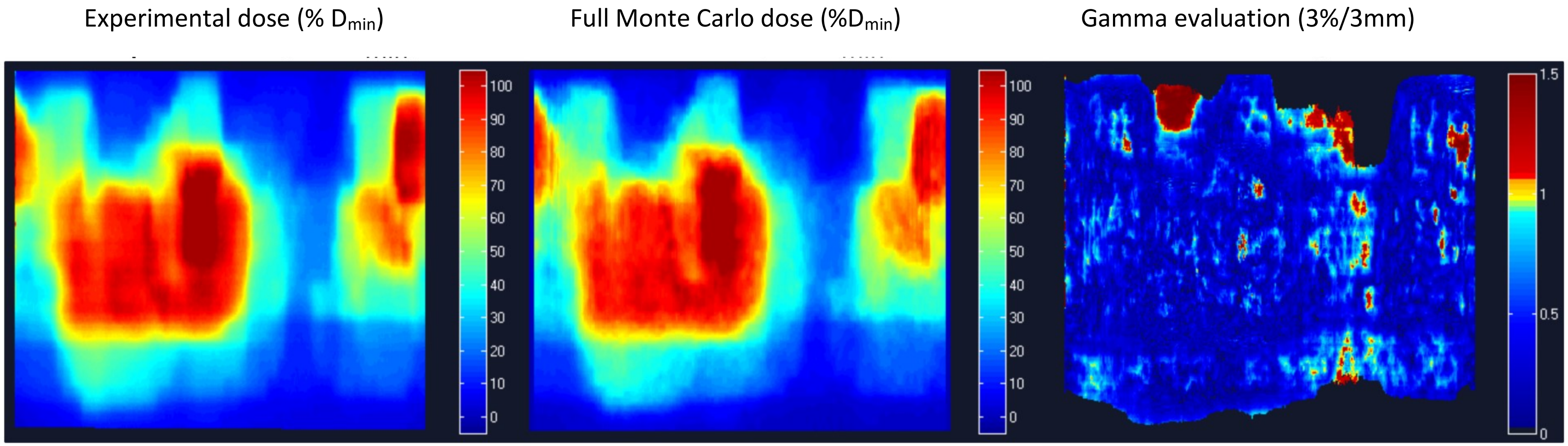

35. Barbeiro AR, Ureba A, Baeza JA, Linares R, Perucha M, Jimenez-Ortega E, et al. 3D VMAT Verification Based on Monte Carlo Log File Simulation with Experimental Feedback from Film Dosimetry. PLoS One 2016;11(11):e0166767.

S1B Figure. Comparison of dose profiles between the same dose distributions of S1A Figure. The position of the dose profiles are shown on both dose distributions. In the lower part, the comparison between horizontal (left) and vertical (right) profiles corresponding to each dose distributions (in red color, the experimental profiles and in green color, the theoretical profiles).

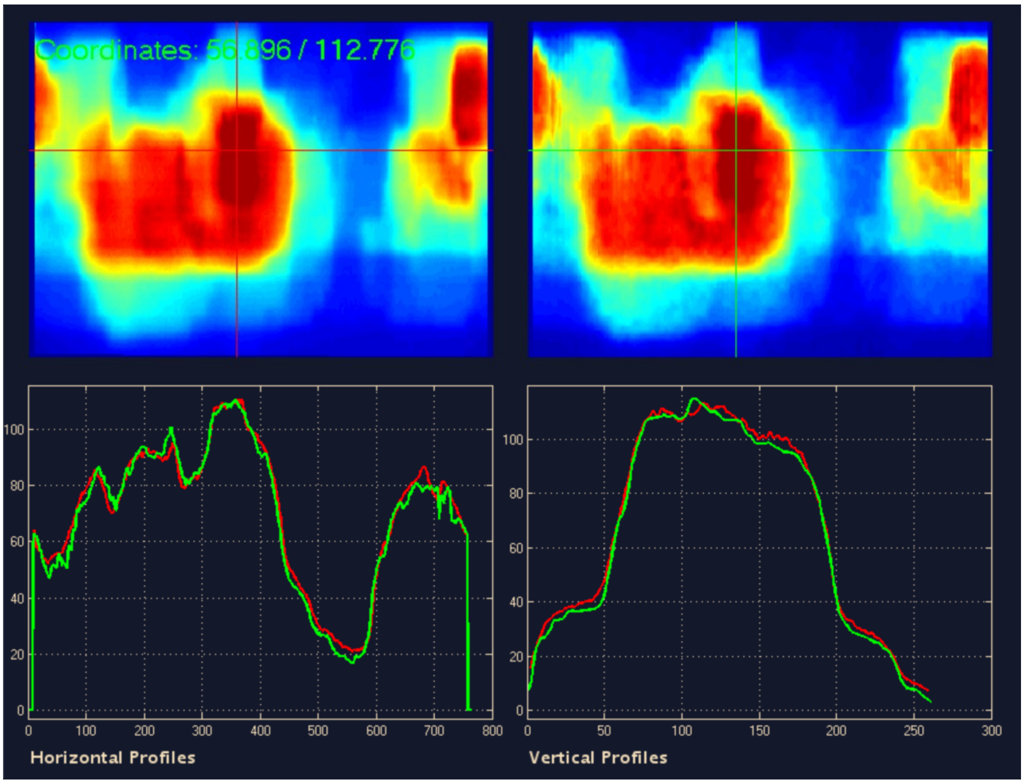

S1C Figure. The same comparison of dose profiles along other directions with sharp gradient.

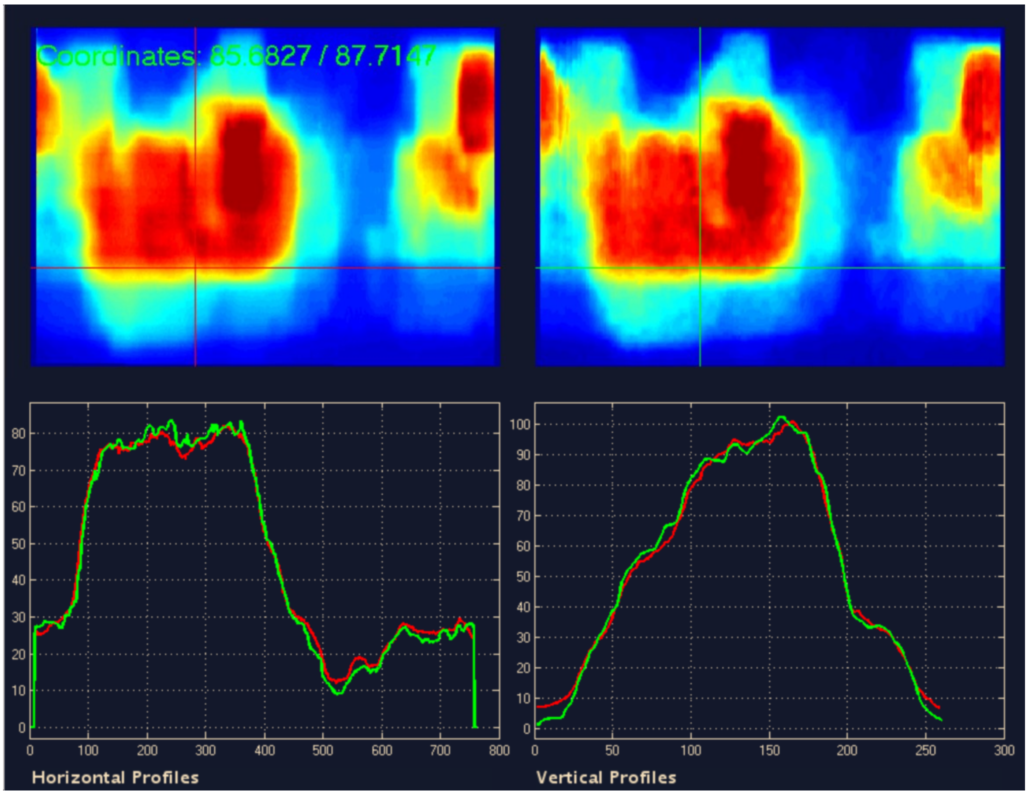

Supplement: S1 Fig — (PDF) [file pone.0210549.s001.pdf]
